# Nutritional Value of Tomato Pomace as a By-Product from the Processing of Several Tomato (*Solanum lycopersicum* L.) Cultivars

**DOI:** 10.3390/molecules30234502

**Published:** 2025-11-21

**Authors:** Anca Becze, Lacrimioara Senila, Mihaela Multescu, Aglaia Popa, Floarea Serbancea, Marin Senila

**Affiliations:** 1 National Institute for Research and Development of Optoelectronics INOE 2000, Research Institute for Analytical Instrumentation, Donath 67, 400293 Cluj-Napoca, Romania; marin.senila@icia.ro; 2 National Institute of Research & Development for Food Bioresources, IBA Bucharest, 6 Dinu Vintila Street, 021102 Bucharest, Romania; mihaela.multescu@gmail.com (M.M.); floarea.serbancea@bioresurse.ro (F.S.); 3 Faculty of Biotechnologies, University of Agronomic Sciences and Veterinary Medicine of Bucharest, 59 Marasti Blvd., 011464 Bucharest, Romania; aglaia.popa@bth.usamv.ro

**Keywords:** tomatoes pomace, fatty acids, metals, carotenoids, lycopene, β-carotene

## Abstract

Tomato processing produces large quantities of by-products, mainly pomace, which are frequently discarded despite their significant nutritional and functional potential. The study aimed to evaluate the proximate analysis, volatiles, fatty acids, total antioxidant capacity, carotenoids, lycopene, and β-carotene in the pomace of three varieties of tomatoes (two Romanian cultivars and one Mediterranean cultivar) for potential valorization of these by-products. The mineral content in the tomato pomace was found to follow the order (average, mg kg^−1^): K (13,090) > P (2793) > Ca (1121) > Mg (879) > Na (258) > Fe (12.3) > Zn (11.4) > Mn (7.0) > Cu (3.0). The fatty acid profiles revealed the presence of linoleic acid, oleic acid, and palmitic acids. All cultivars exhibited a high content of polyunsaturated fatty acid (PUFA) (55.7–62.3%) and a low content of omega-3 fatty acids (2%). Nutritional quality indices were calculated based on the fatty acid profiles. Analysis of volatile compounds revealed that Mediterranean cultivars were predominantly enriched in alcohols, hydrocarbons, and aldehydes, whereas Romanian cultivars had a high level of terpenes, along with hydrocarbons and aldehydes, contributing to their distinctive flavor profiles. The identified terpenes included β-phellandrene, terpinolene, limonene, ocimene, and cholestanone. All samples exhibited notable levels of bioactive compounds, particularly polyphenols and carotenoids. Additionally, high quantities of lycopene were found in the tomato by-products. The nutritional quality indices indicate that tomato pomace can be utilized as an ingredient to enhance the nutritional value of other products.

## 1. Introduction

Tomatoes (*Solanum lycopersicum* L.) are a highly nutritious vegetable used worldwide and possess significant economic value. During juice production, a substantial amount of waste is generated. Tomato pomace, including peels and seeds, is produced as a by-product in the food industry. This byproduct contains dietary fiber (cellulose and hemicellulose (39–59%)), pigments (lycopene and β-carotene), phenolic compounds, tannins, fatty acids, organic acids, vitamins, proteins, and many other bioactive and health-promoting components. The peel is rich in dietary fiber and lycopene, and the seeds contain high levels of oil and protein [1]. The amount of tomato by-products constitutes approximately 3–5% (*w*/*w*) of raw tomatoes [2]. The content of polyphenols and dietary fiber varies depending on the type of fruit, geographical origin, and extraction method used. The primary bioactive compounds include carotenoids, polyphenols, vitamins, proteins, and fatty acids. This waste can be valorized through the production of nutraceuticals (such as the extraction of lycopene and antioxidants), food additives, fermentation feedstock, compost, biochar, and packaging films or biomaterials. It was estimated that up to 50% of vegetables and fruits are lost as waste during their processing, without being used for other purposes [3]. Tomato pomace contains a high moisture content, ranging from 64.31% to 92.55%. Specifically, fresh tomato pomace has a moisture content of 87.63%, while freeze-dried pomace contains 7.37%, and cabinet-dried pomace has 4.77% [4]. The proximate composition of dried tomato pomace has been reported as follows: moisture 5%, ash 3.5%, protein 32.6%, carbohydrates 43.3%, and fiber 29.4%. Tomato pomace is rich in amino acids, including aspartic acid, glutamic acid, arginine, leucine, isoleucine, and glycine. Additionally, tomato pomace contains vitamins, among them vitamin B12 and vitamin C. The following minerals have been reported (µg g^−1^): calcium (146.4), potassium (2686.9), iron (29.2), magnesium (281.3), and sodium (210.3). Regarding flavonoids, the following compounds have been identified: epicatechin, quercetin, and rutin. The phenolic acids reported include coumaric acid, caffeic acid, chlorogenic acid, gallic acid, syringic acid, vanillic acid, trans-cinnamic acid, ellagic acid, and ferulic acid [5].

Tomato peel by-products contain a significant amount of carotenoid and terpene pigments, which are responsible for the color of tomatoes. Carotenoids can be utilized in numerous applications, including food supplements, nutraceutical ingredients for food and feed products, and the production of cosmetic products [6]. Among these, tomato by-products contain a significant amount of lycopene (C_40_H_56_). Lycopene exists in two forms: *cis* and *trans*, with approximately 90% being in the trans form and 10% in the *cis* form. There are 72 geometric configurations of lycopene, including E-lycopene, 5Z-lycopene, neolycopene A (6-*cis*-lycopene), prolycopene (1-, 3-, 5-, 7-, 9-, 11-*cis*-lycopene), and *cis*-lycopene (1-, 3-, 5-, 6-, 7-, 9-, 11-*cis*-lycopene) [7]. Marinaccio et al. [7] reported a method for extracting lycopene from tomato skin waste using ultrasound-assisted techniques with three solvents: hexane, α-pinene, and a mixture of thymol and menthol. The highest extraction yield was achieved with hexane as the solvent, resulting in 735.9 mg/g of dried extract [7]. Phenolic compounds are present in tomato peels, including caffeic, chlorogenic, p-coumaric, ferulic, and rosmarinic acids, as well as flavonoids (quercetin and rutin), and have nutraceutical potential due to the antioxidant and antiproliferative activities of the extract. These byproducts can be revalorized as functional ingredients in the context of a circular economy. The Farm to Fork (F2F) strategy includes reducing pesticide use by 50% and replacing it with biopesticides such as terpenes, phenolic acids, stilbenes, tannins, alkaloids, nematicides, fungicides, and insect growth regulators [8]. The volatile organic compound is affected by genetic information, environmental conditions, post-harvest management and storage, and degree of maturity. During maturation, many processes like structural changes, starch degradation, sugar production, synthesis of pigments occur and produce esters, alcohols, aldehydes, ketones, and terpenoids [9]. Several authors have reported the presence of the following fatty acids: linoleic acid (C18:2) at 54%, oleic acid (C18:1) at 22%, linolenic acid (C18:3) at 2%, and palmitoleic acid (C16:1) at 0.3%. Although tomato pomace oil has a lower fatty acid content than sunflower and soybean oils, its richness in linoleic and oleic acids makes its composition comparable to edible oils, suggesting it could be a promising source in the future [10]. Numerous studies have reported the use of tomato pomace in dietary supplementation as a valuable functional ingredient for improving the physicochemical and sensory properties of foods [11]. An additional research group prepared a gluten-free snack using potato flour and tomato pomace [12]. The experimental findings demonstrated that the incorporation of tomatoes into the product formulation led to a substantial enhancement in the texture and hardness of the final product. Concurrently, the addition of tomato pomace resulted in a reduction in oil content, accompanied by a concomitant increase in fiber. Another study reported the use of tomato pomace to obtain pomace powder/polylactic acid foams in order to improve foaming behavior and to create sustainable metal food packaging, as well as a novel corrosion inhibitor for steel in industrial environments [13]. Mineral elements play significant roles in maintaining physiological functions of the human body. These influence blood pressure, muscle contraction, nerve transmission; regulate metabolic pathways and pH balance of the human body; participate in the creation of vital organs; and contribute to the energy production [14]. However, even if about three-quarters of the known elements are metals, only several are essential for humans [15]. According to several studies [11], essential elements found in tomatoes are potassium (K), calcium (Ca), phosphorus (P), magnesium (Mg) as major minerals and iron (Fe), copper (Cu), zinc (Zn), sodium (Na), manganese (Mn), and boron (B) as minor minerals. Some non-essential elements like cadmium (Cd), lead (Pb), mercury (Hg), arsenic (As), or aluminum (Al) have no known biological role, and are toxic even at very low concentrations [16,17]. Among the essential elements, K, P, Mg, Ca, Na, and Fe are abundantly found in tomato peels and seeds [11,18,19,20,21,22].

The chemical characterization of these by-products is essential to identify their potential as sustainable sources of functional ingredients for the food industry. Variations in cultivar, growing region, and genetic background can significantly influence the nutritional and phytochemical profiles of tomato seeds, affecting their suitability for valorization.

Proximate analysis refers to the standard set of compositional determinations used to provide a rapid estimate of the main nutritional constituents of a sample. Typically, proximate analysis includes measurements of moisture, ash (mineral content), crude protein, crude fat, crude fiber, and carbohydrates. This set of analyses is widely used to characterize the overall chemical composition of plant-derived materials and to compare compositional changes resulting from treatments or processing.

This study not only facilitates the development of value-added food products but also contributes to reducing agro-industrial waste and promoting a circular economy. The Romanian cultivars are widely grown in local agricultural systems because of their high yield potential, adaptability to temperate climates, and resistance to common fungal and bacterial pathogens. These cultivars are especially valued for their seed-rich by-products, which offer significant opportunities for valorization. The Mediterranean cultivar has grown under different climatic conditions, including arid and warmer environments, as well as varying soil characteristics. Under these conditions, its chemical composition has different properties. In this context, a comparative evaluation of the nutritional and chemical characteristics of both cultivars will be valuable for the development of value-added products from tomato by-products. The first objective of this study was to evaluate the chemical composition of by-products derived from three different tomato cultivars, focusing on proximate analysis, mineral content, bioactive compounds, antioxidant capacity, fatty acid profiles, and volatile compounds, with the aim of utilizing these by-products as ingredients in other food products. The secondary objective was to calculate nutritional quality indices based on fatty acid profiles to determine their potential effects on human health.

## 2. Results and Discussion

### 2.1. Proximate Analysis of Pomace By-Products

The nutritional composition of the pomace fractions from three tomato cultivars, *Olteana F1* (R1), *Trandafirii de Buzau* (R2), and *Macizo F1* (R3), is presented in Table 1.

These fractions were analyzed for dry matter, fat, protein, ash, carbohydrates, and total sugars. Among the cultivars, R3 exhibited the highest dry matter content (34.74 ± 4.41%), followed by R1 (33.31 ± 3.34%) and R2 (33.14 ± 2.71%). However, the differences were not statistically significant (*p* > 0.05), indicating a relatively uniform water content across cultivars, which supports their potential for consistent behavior during industrial processes such as drying or extrusion. Fat content showed only slight variation, ranging from 1.7 ± 0.17% in R3 to 2.4 ± 0.25% in R1. Tomato pomace of the R1 cultivar had the highest average lipid content, which was, however, significantly different (*p* > 0.05) from that of the R2 and R3 cultivars. The values are slightly lower than typical values found in pure tomato seeds (14–25%) but consistent with mixed peel–seed fractions or peel alone (1–3%) reported by Sánchez-Moreno et al. [23]. Protein content, however, varied more noticeably. R2 recorded the highest level (8.43 ± 0.69%), significantly higher (*p* < 0.05) than R1 (6.66 ± 0.63%) and R3 (7.14 ± 0.58%). The values align with previous reports of 5–10% protein in tomato pomace residues [24,25]. These findings highlight the potential of R2 as a superior source of plant protein among the studied cultivars. Ash content, reflecting mineral load, was in the range of 0.88 ± 0.11% in R2 to 1.33 ± 0.14% in R3. These results are lower than some values found in tomato peel alone (typically 2.5–4%), likely due to dilution in the peel–seeds matrix or cultivar-specific differences [24]. Although the numerical difference is apparent, statistical comparison was limited due to censored data in two groups. Dietary fiber content ranged from 7.27 ± 0.71% in R2 to 9.25 ± 0.85% in R3, with R1 showing an intermediate value of 7.97 ± 0.71%. These values are consistent with literature reports for tomato peel and seed by-products, which typically contain between 7 and 12% fiber on a fresh weight basis, depending on cultivar and processing conditions [24,25,26]. Unlike crude fiber, which captures only a limited portion of indigestible cellulose and lignin, dietary fiber encompasses both insoluble (e.g., cellulose, hemicellulose) and soluble fractions (e.g., pectin, gums), contributing to important physiological functions such as improved digestive health and glycemic control. The significantly higher fiber content in R3 (*p* < 0.05) suggests greater potential for use in functional food formulations or as a dietary supplement in feed, particularly for its water-holding and fermentability properties [27]. Carbohydrates were present in substantial amounts across all cultivars, with R1 showing the highest content (32.7 ± 3.30%), followed by R3 (26.5 ± 2.74%) and R2 (26.0 ± 2.36%), similar to value found by Kaboré et al. [26]. R1 tended to have a higher carbohydrate content (32.7 ± 3.30%), which was significantly greater (*p* < 0.05) than that of R2 and R3. Notably, R2 also had the highest total sugar content (6.7 ± 0.61%), followed by R1 (5.1 ± 0.52%) and R3 (5.0 ± 0.55%). The difference in sugar content between R2 and the others was statistically significant (*p* < 0.05), suggesting greater fermentative potential and possibly enhanced palatability. This range aligns well with literature values for reducing and total sugars in tomato peels (4.5–7.5%), as reported by Anđelini et al. [28], and indicates a high potential for microbial fermentation or flavor enhancement in food applications.

### 2.2. Mineral Elements in Tomato Pomace

The contents of mineral element in tomato pomace are presented in Table 2.

In general, the mineral content in tomato pomace decreases in the order K > P > Ca > Mg > Na > Fe > Zn > Mn > Cu, as reported in several previous studies [18,19,20,21,22,29,30,31], tomato peels and seeds are rich in valuable minerals that are important for the human diet. The contents of K in the pomace of the three tomato varieties were 11,940 ± 1020 (R1), 13,130 ± 1220 (R2), and 14,200 ± 1230 mg kg^−1^ dw (R3). These values agree with the range of 10,970 to 45,830 mg kg^−1^ reported by Chabi et al. [10] for K in tomato peels. K is an essential element in humans, present in all tissues, and is vital for normal cell function with a role in maintaining electrochemical gradients in cell membranes [32,33]. It has a strong relationship with Na, which is a key regulator of extracellular fluid volume. However, since high Na intake is associated with heart diseases, including hypertension, the fact that Na content (166 ± 18 to 385 ± 45 mg kg^−1^ dw) in tomato peels is more significant than that of K represents an advantage. The sodium content measured in our study was slightly lower than that reported by Chabi et al. [11] (720–783.3 mg kg^−1^ dw). Manganese is also an essential element with a role in amino acid, glucose, cholesterol, and carbohydrate metabolism. Copper contributes to the production of red blood cells, and the health of the immune system, bones, blood vessels, and nerves [34].

Even if found in lower amounts, other essential elements such as Fe, Mn, Cu, and Zn are present in tomato pomace. Iron is well-known for its role in hemoglobin, the protein that transports oxygen in blood [35]. Zinc is another essential element for the human body, involved in immune system, protein, and DNA production, supporting healthy growth and development [35].

Phosphorus (P) is another abundant element in tomato peels. This vital mineral is essential for maintaining strong bones, together in conjunction with calcium (Ca). This is also a component of DNA and RNA, and is a key element in producing energy through adenosine triphosphate (ATP) [31]. The content of P found in tomato peels in our study was between 2461 ± 185 and 2990 ± 310 mg kg^−1^ dw, and displayed low variabilities among different tomato species. A similar range of P content (2620 to 3430 mg kg^−1^ dw) was previously reported [10]. Another major element found in tomato peels was Mg, in the range of 765 ± 110 to 981 ± 137 mg kg^−1^ dw. Our findings showed Mg levels slightly lower than those reported in previous studies for tomato peels (1356–2656 mg kg^−1^). Nevertheless, tomato pomace remains an important source of Mg. Magnesium is a cofactor in over 300 enzymes with important roles in human body, including in muscle and nerve function, blood sugar and blood pressure control, and energy production [36].

### 2.3. Lipid Content, Fatty Acids

The lipid content was 2.4% in cultivar R1, 1.9% in cultivar R2, and 1.7% in cultivar R3. The fatty acid identified in tomato pomace as a by-product was presented in Table 3.

The varieties of tomato peels contain saturated (SFA), monounsaturated (MUFA), and polyunsaturated fatty acids (PUFA). The saturated fatty acids present include myristic acid (C14:0), palmitic acid (C16:0), heptadecanoic acid (C17:0), stearic acid (C18:0), arachidic acid (C20:0), behenic acid (C22:0), and lignoceric acid (C24:0). The primary saturated fatty acids found in tomato pomace are palmitic acid (C16:0) and stearic acid (C18:0). Variety R2 exhibits the highest concentration of palmitic acid (C16:0), along with a slow increase in behenic acid (C22:0) and stearic acid (C18:0). The content of SFA is low in all varieties. A high concentration of SFA has been demonstrated to have the potential to contribute to low-density lipoproteins (LDL) cholesterol in blood serum. The highest concentration was determined for C16:0. Oleic acid (C18:1), non-essential fatty acid, is found in moderate concentrations in all varieties, contributing to the monounsaturated fatty acid (MUFA) content, particularly to the omega-9 family. According to Tutunchi et al. [37], oleic acid is recognized for its resistance to oxidation (compared with PUFA). The impact of oleic acid on cardiovascular health remains unclear, but linolenic acid is an essential fatty acid that must be obtained from diet [37]. The MUFA content is highest in R3 variety, indicating a favorable lipid profile due to its cardioprotective effects. Linoleic acid (C18:2(n-6)) is the predominant fatty acid found in all varieties, with a content ranging from 45.19% in R3 variety to 55.14% the R1 variety. In our work, *cis* and *trans* isomers were quantified together. The similar results were reported by Rodríguez et al. [10]. Variety R1 has the highest PUFA content (62.30 ± 6.35^a^ %), particularly linoleic acid, suggesting a rich omega-6 profile. All variety samples are abundant in omega-6, indicating a strong pro-inflammatory potential [38], and it is recommended to balance this with omega-3. Unsaturated fatty acids (UFAs) exceed 85% in all samples. Unsaturated fatty acids contain omega-9, omega-6, and omega-3, depending on the number of double bonds. Low quantities of omega-3 were detected in all varieties and were around 2%. The findings of this study are in concordance with those previously reported by Rodríguez et al. [10] and Pérez et al. [39]. These studies analyzed the nitration of fatty acids in tomato pomace as a method of combating cardiovascular disease. The resulting products were incorporated into the diet with a view to enhancing the antiplatelet potential by introducing bioactive lipids from tomato pomace for the treatment of cardiovascular disease and thrombosis.

### 2.4. HS-SPME GC-MS Analysis of Volatile Organic Compounds

The GC-MS analysis by using headspace solid-phase microextraction (HS-SPME) identified 57 volatile organic compounds (VOCs). Table 4 shows the identified compound, retention time, relative percentage content (represents a measure of the contribution of each peak to the total chromatogram), sensory descriptor, and compound class. The VOCs identified in pomace, including hydrocarbons, terpenoids, aldehydes, ketones, esters, alcohols, nitrogenous compounds, amines, amides, nitriles, isocyanates, pyridine, and pyrazine, contribute to the aroma. Hydrocarbons found in R1 cultivar are as follows: 1,5-hexadiyne, 3-methyl-2,4-hexadiene, propylcyclopropane, 1-butene, (E)-1,2-dimethylcyclopropane, 1-pentene, n-methylene-ethenamine, and 1-fluoroheptane, whereas in sample 2 the following compounds are found: 4-methylcyclohexene, 5-chloro-3-methylpenta-1,3-diene, 1,3-butadiene, 2-vinylbicyclo [2.1.1]hex-2-ene, 2,4-octadiyne, propylcyclopropane, 1-butene, 1,5-heptadien-3-yne, 3,7,7-trimethylcyclohepta-1,3,5-triene, 2-methyl-1,3-pentadiene, and 3-methyl-4-methylenebicyclo(3.2.1)oct-2-ene. Hydrocarbons present many VOCs and had the highest proportion and induced green, herbaceous, slightly sweet, pungent, and fruity odors. Trans-1,4-hexadiene, present only in the R2 variety (19.8%), imparts a light green and slightly sweet aroma.

Aldehydes are derived from the oxidative degradation of unsaturated fatty amino acids or the hydrolysis of triglycerides and are readily oxidized upon exposure to air. These specific aldehydes contribute green, herbal, and fresh flavor notes. Variety R1 contains a high content of hexenal (6.96%) and 2-hexenal, whereas sample R2 is rich in terpenes (beta-phellandrene, terpinolene, limonene) and some hydrocarbons. This composition indicates lipid oxidation and gives a strong aroma characterized by sweet, green, herbal, fruity, woody, and citrus notes. At very low concentrations, hexanal induces the trace of freshly cut grass and unripe fruits, particularly apples and plums. At higher concentrations, its sharp, acidic notes become more pronounced, resembling the rancid flavor of spoiled butter. Variety R3 contains 1-hexanol, methylvinylketone, furans, and pyrazines, which are also found in significant amounts in variety R1. Hexanal is an aldehyde commonly present in various fruits and vegetables, contributing to a grassy and green aroma. Hexanal is produced through the decomposition of polyunsaturated fatty acids (PUFAs), particularly linoleic acid, into aldehydes. The initial step involves the oxidation of PUFAs to produce oxylipins, a process catalyzed by lipoxygenase, which ultimately leads to its formation. This compound is widely utilized in the food and cosmetic industries [40].

Terpenes and terpenoids are present in high quantities in tomato pomace varieties.

Variety R2 contains terpenes such as terpinolene (20.05 ± 1.98%), limonene (1.57 ± 0.14%), ocimene (0.52 ± 0.03%), and β-phellandrene (47.23 ± 4.1%). Cholestanone was detected only in variety R3 (0.47 ± 0.05%). According to Mahizan et al. [41], terpinolene acts as a natural antioxidant and has antimicrobial properties. Terpinolene was found only in variety R2 (20.05%) and gives the fresh, herbal, and green aroma. Limonene was detected in low amounts and gave citrus, pine, and fresh lemon aromas and was detected in variety R2. Limonene is a monoterpenoid used as solvent for rosin, waxes, rubber, and other applications. β-Phellandrene is a cyclic monoterpene that is found in high quantities (47%) and has antimicrobial and anti-inflammatory properties. It was detected only in the variety R2.

Pizzo et al. [42] reported that *β*-phellandrene was the predominant monoterpene found in tomato leaf extracts, as well as the result of terpene synthase in tomatoes. It is the main volatile compound in the monoterpene class and is found in plant leaves, giving them a minty, light citrus, green, and herbal aroma. Tomato plants produce a variety of terpenes within their trichomes. Terpenes and terpenoids are a diverse family of compounds found across many plants, animals, and microorganisms. They have distinctive odors and play a crucial role in plant chemical defense, acting as repellents or toxins against herbivores and pathogens, while also attracting the natural enemies of these threats [42]. Terpenes give the aroma of citrus, sweet, fresh, and fruity odor, whereas terpinolene gives the piney, sweet, earthy with notes of citrus.

Prenol is found naturally in fruit (grapes, apples, and other fruits) and is used in the biosynthesis of terpenes. The 3-hexenol is known as “leaf alcohol” and is found in numerous plants, contributing to fresh aroma. Prenol was found only in variety R2 (4.74%).

Alcohols found in tomato pomace are 1-hexanol, 2-methyl-1-propanol, and 1-Octen-3-ol, and they give the green, grassy, fresh, and alcoholic taste. 1-hexanol and 1-octen-3-ol are derived from oxidation of linoleic acid, whereas 2-methyl-1-propanol is derived from valine metabolism, decarboxylation of α-ketoisovalerate, a valine metabolite, followed by reduction [43]. 1-Hexanol, which contributes to the food’s aroma, was detected in high quantities only in the R3 variety (30.91%). It has also been reported to be present in fresh tomatoes and tomato sauces [44].

Ketones are present in lower amounts, including methylvinylketone, 6-methyl-5-heptene-2-one, and cyclohexane-1,3-dione, as well as 2-allylaminomethylene-5,5-dimethyl. The latter is a dione recognized as a precursor for the synthesis of bioactive compounds [45]. While ketones can produce a pungent or acrid odor in isolation, they often impart fruity, green, and herbal aromas when combined. 2-penthylfuran was the only furan found in R1 and R3 varieties. The presence of heterocyclic compounds is in low quantities. Azetidine was found only in varieties R2 and R3 and gives the ammoniacal, fishy, pungent taste [44]. In small amounts, two nitrogen compounds were detected and contribute to the pungent, sharp, and slightly sweet aroma.

### 2.5. Bioactive Compounds and Antioxidant Capacity

The pomace of the analyzed tomato cultivars exhibited notable levels of bioactive compounds, particularly polyphenols and carotenoids, along with a moderate antioxidant capacity (Table 5).

The total polyphenol content (mg GAE.kg^−1^) ranged from 122.03 ± 10.0 in R2 to 134.71 ± 12.3 in R1, with R3 presenting a similar concentration (133.85 ± 11.5). Although R1 showed the highest average polyphenol content, the differences among cultivars were not statistically significant (*p* > 0.05), as the standard deviations overlapped across samples. These values fall within the lower end of the range reported for tomato peels and seeds in the literature, where total phenolic content may vary from 120 to over 500 mg GAE kg^−1^, depending on cultivar, drying method, and extraction protocol [8,46]. The antioxidant capacity, assessed as µg Trolox equivalents per gram, was similar in R1 and R3 (both 0.34 ± 0.03), and slightly lower in R2 (0.31 ± 0.03), again with no statistically significant variation observed between groups (*p* > 0.05). These values are comparable with those reported in tomato pomace extracts, which typically range from 0.28 to 0.55 µg eq Trolox. g^−1^ [46,47]. In terms of carotenoid profile, R1 stood out with the highest total carotenoid content (4.24 ± 0.40 mg eq. β-carotene. g^−1^), significantly higher (*p* < 0.05) than that of R2 (2.24 ± 0.22 mg eq. β-carotene. g^−1^). The β-carotene levels ranged from 2.95 ± 0.27 µg g^−1^ to 3.14 ± 0.30 µg g^−1^, while lycopene content varied more distinctly, with R3 registering the highest value (7.41 ± 0.68 µg g^−1^), followed by R1 (6.22 ± 0.63 µg g^−1^) and R2 (6.10 ± 0.56 µg g^−1^). The β-carotene levels ranged from 2.95 ± 0.27 µg g^−1^ to 3.14 ± 0.30 µg g^−1^, values similar to those reported for tomato peel by-products (typically 2–4 µg g^−1^) [8,46]. Lycopene content varied more distinctly, with R3 registering the highest value (7.41 ± 0.68 µg g^−1^), followed by R1 (6.22 ± 0.63 µg g^−1^) and R2 (6.10 ± 0.56 µg g^−1^). These levels are within the expected range reported for tomato waste fractions, often cited between 5 and 10 µg g^−1^ [46,48]. Despite these apparent differences, statistical significance was not always observed due to relatively high intra-sample variability. Overall, the results highlight that tomato pomace retains a relevant quantity of antioxidant compounds post-processing, supporting their valorization as functional ingredients in food or feed applications.

### 2.6. Nutritional Quality Indices

Nutritional quality indices of tomato pomace are shown in Table 6.

As illustrated in Table 6, the calculated nutritional indices for the tomato pomace variety are presented. The mean MUFA/SFA ratio was found to range from 1.76 (R2) to 2.34 (R3). As stated in Kenneth et al. [49], a ratio greater than 1 is advantageous to one’s health, with cardiovascular problems being the most prevalent concern. With regard to the PUFA/SFA ratio, it is evident that in all cases it exceeds 0.45 (the value recommended by the UK Department of Health). This is attributable to the high concentration of PUFA in all varieties. According to Maki et al. [50], limiting the intake of SFA to below 10% of energy/day and replacing with UFA. However, increasing PUFA intake is controversial, since high consumption of omega-6 PUFAs may be problematic, and maintaining a balanced ratio of ω-6 to ω-3 fatty acids is recommended. The predominant ω-6 PUFAs are linoleic acid (LA, 18:2, ω-6 FA) and gamma-linolenic acid (GLA, C18:3, ω-6 FA), and they have anti-inflammatory effects.

The ratio of omega-6/omega-3 is between 27.3 and 18.17. The optimum ratio should be 2:1 to 10:1, depending on the source [50]. The observed ratio is considerably higher, and it is therefore recommended to reduce omega-6 intake and/or increase omega-3 intake in order to achieve a more balanced and health-promoting profile.

Polyunsaturated fatty acids have a significant role in cardiovascular health. Atherogenicity (AI) and thrombogenicity (TI) indices are commonly used to assess the potential health benefits of specific foods. The AI and TI values were low in all samples, suggesting that consumption of these foods could have beneficial effects in reducing thrombus and plaque formation in blood vessels, thereby supporting heart health. A lower AI is recommended for a healthy diet, as a high AI value is associated with an increased tendency to form clots in blood vessels and promote platelet aggregation. It is advisable to consume foods with an AI below 1 and a TI below 0.5. These limits are encountered by tomato pomace [51].

NVI values were higher in all samples, indicating a high nutritional potential. The potential for lipid oxidation is significant, as lipids generally have the potential for oxidation, which can lead to reduced oxidative stability. This stability is an important indicator for food storage and processing.

Desired fatty acids are defined as the sum of stearic acid and UFA, providing information regarding the hypocholesterolemic characteristics of lipids, which help lower total cholesterol levels. The DFA index is higher due to the high PUFA content, making these by-products suitable for dietary purposes [52].

The hypocholesterolemic and hypercholesterolemic fatty acids index (h/H) evaluates the effects of hypocholesterolemic and hypercholesterolemic fatty acids on cholesterol levels, with a lower index being desirable (below 1). The results obtained showed an h/H index higher than 5, indicating that tomatoes are considered nutritionally rich for human consumption, but should be consumed in lower quantities [52].

Unsaturated fatty acids and MUFA are recognized as hypocholesterolemic fatty acids that are beneficial for human health and are utilized in cholesterol management. The hypocholesterolemic potential index is linked to the UFA content in tomato pomace and may help reduce blood cholesterol levels. This relationship is associated with lower values of AI (atherogenic index), TI (thrombogenic index), and HPI.

Tomato pomace, a byproduct of processing tomato juice and paste, is a valuable source of bioactive compounds, dietary fiber, and natural antioxidants. Its potential health benefits and cardioprotective effects are due to essential fatty acids, carotenoids, and polyphenols. Technologically, incorporating tomato pomace as a functional ingredient can improve the nutritional quality of food products by enhancing fiber content, color, and natural preservative properties due to its antioxidant activity. The valorization of tomato pomace supports sustainable food processing practices and is a cost-effective source of bioactive compounds that have technological benefits.

### 2.7. Statistical Analysis

**Figure 1:**
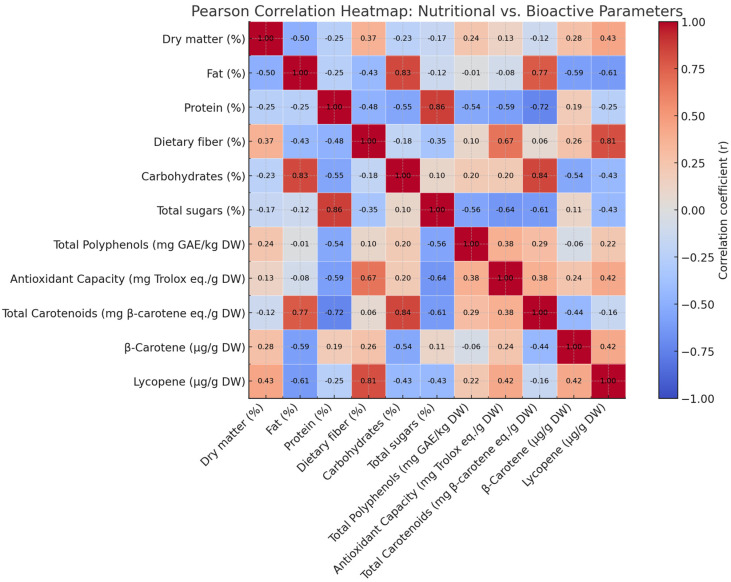
Heatmap representing Pearsons’s correlation between nutritional and bioactive compounds.

A Pearson correlation heatmap was generated to evaluate potential linear relationships among the nutritional and bioactive compounds quantified in the analyzed tomato cultivars. As shown in Figure 1, several noteworthy trends emerged.

Dietary fiber showed a moderate positive correlation with total polyphenol content (r ≈ 0.48), indicating a potential co-localization or co-extraction effect, whereby fiber-rich matrices enhance the retention or stability of phenolic compounds. This relationship supports the role of plant cell wall components in modulating antioxidant capacity. Conversely, total sugar content demonstrated a weak negative correlation with antioxidant capacity and total polyphenols. Although not statistically strong, this trend may suggest that cultivars with higher sugar levels possess comparatively lower concentrations of antioxidant phytochemicals, possibly due to metabolic trade-offs. Lycopene content, a key carotenoid, showed weak correlations with most macronutrient parameters, indicating its biosynthesis may be regulated independently of major nutritional components. Overall, the correlation analysis provides valuable insight into the compositional interrelationships within tomato matrices and supports the strategic selection of cultivars with enhanced functional and nutritional profiles.

**Figure 2:**
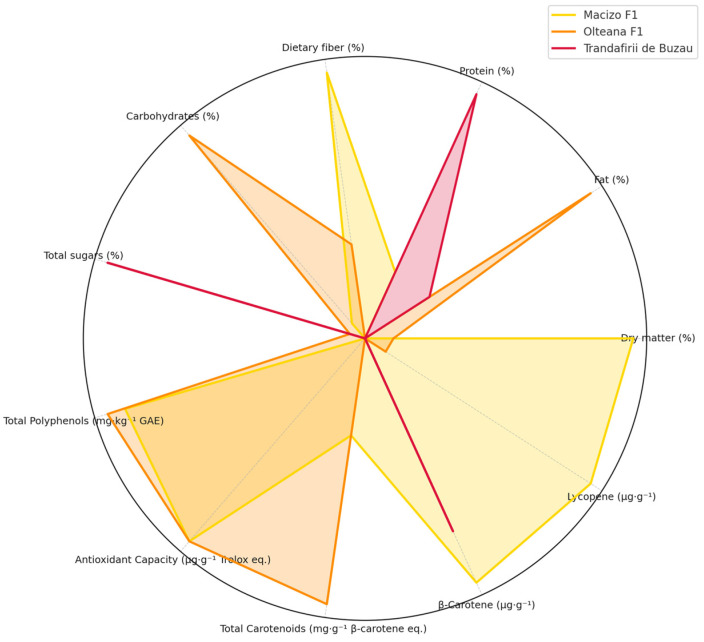
Multivariate analysis on the compositional differences and similarities of tomatoes varieties.

To provide a comprehensive visual comparison of the nutritional and bioactive characteristics among the studied tomato cultivars, a radar chart was constructed based on normalized mean values (Figure 2). This multivariate graphical representation highlights key compositional differences and similarities across 11 parameters, including macronutrients (e.g., protein, fat, dietary fiber), as well as functional compounds such as total polyphenols, carotenoids, β-carotene, and lycopene.

The radar chart reveals distinct compositional profiles for each cultivar. The R1 variety exhibited elevated levels of dry matter, total polyphenols, and total carotenoids, indicating its potential as a high-value functional food source. The R1 variety may be preferred as a source of carotenoids for supplements, given its higher total carotenoid content and favorable profile of bioactive compounds, which could enhance its nutritional and functional value in health-oriented formulations.

R2 showed comparatively higher β-carotene content and antioxidant capacity, suggesting a more pronounced provitamin A and radical-scavenging potential. In contrast, R3 presented a balanced distribution across most parameters, with moderate levels of both nutritional and bioactive constituents. This visualization underscores the complementary strengths of the cultivars and supports their differential use in food formulations or breeding programs targeting enhanced nutritional or functional quality. The radar plot proves particularly effective in summarizing complex datasets and facilitating cultivar selection based on multiple compositional criteria.

**Figure 3:**
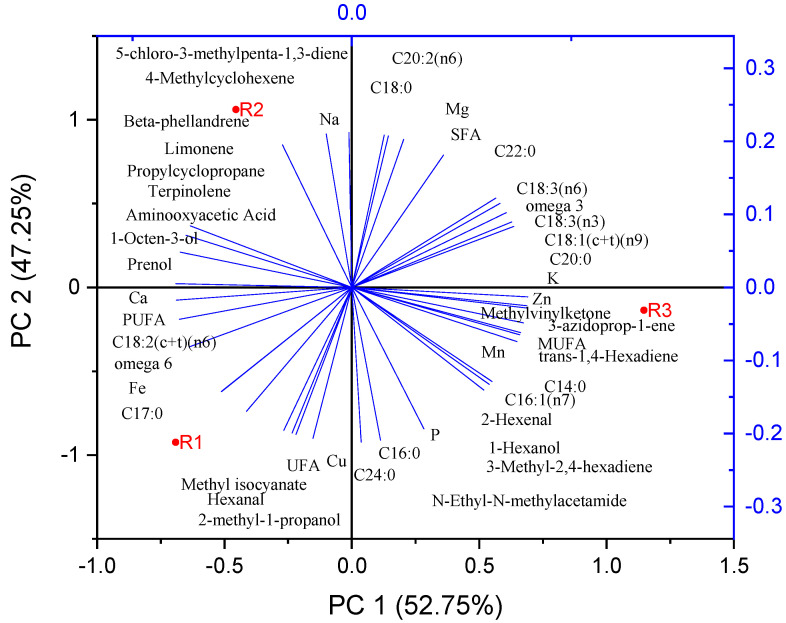
Principal component analysis and relationship among fatty acids, metals, and some volatile parameters.

In Figure 3 is presented the principal component analysis and relationship among fatty acids, metals, and some volatile parameters.

The observed differences in nutritional and mineral content among the R1, R2, and R3 cultivars can be attributed to a combination of genetic and environmental factors. The R1 and R2 cultivars, both of Romanian origin, have similar profiles; however, R1 has higher levels of PUFAs (notably C18:2), UFA, and minerals such as Ca, Fe, and Cu. These differences may result from cultivar-specific genetic regulation of lipid metabolism and mineral uptake. The presence of terpinolene and elevated Na levels in R2 reflect distinct enzymatic pathways and ion transport mechanisms characteristic of this cultivar. R3, of Mediterranean origin, shows elevated Zn, Mn, P, MUFAs, SFAs, and a broader range of VOCs, suggesting genetic adaptation to different environmental conditions, including soil composition, climate, and nutrient availability, which influence both primary and secondary metabolism [52]. Similarities across the three cultivars, such as fatty acid classes and certain VOCs, indicate conserved metabolic processes inherent to tomato seeds. Overall, the phytochemical profiles of tomato by-products are crucial for their valorization in food applications.

## 3. Materials and Methods

### 3.1. Tomato Samples and Preparation of Tomato Pomace as By-Products

Three tomato cultivars, Olteana F1 (*Solanum lycopersicum* L.) (R1), Trandafirii de Buzău (R2), and Macizo F1 (R3), were selected for analysis. R1 and R2 have Romanian origin, whereas R3 has Mediterranean origin. These samples, harvested in 2025, were obtained from different retail locations across Romania, ensuring representative sourcing of commercially available products. The three tomato cultivars (two Romanian and one Mediterranean) were intentionally chosen to capture genetic and geographical diversity. The Romanian cultivars (Olteana F1, Trandafirii de Buzău) represent locally dominant types used in national processing industries, while Macizo F1 represents an international reference. This selection ensures variability in environmental and genetic backgrounds, which is essential for comparative nutritional and phytochemical assessment. All samples were retail-sourced from producers adhering to national agricultural standards and verified origin, which provides representative consumer-level samples rather than experimental field samples. The use of market-available tomatoes reflects realistic by-product variability encountered in the food industry and thus strengthens the applicability and translational relevance of the results. All cultivars were grown under Romanian agricultural conditions. The study included nine biological replicates per cultivar (n = 9), ensuring sufficient statistical power and reproducibility. The samples were transported under refrigeration and stored at 4 °C until processing, which was carried out within 24 h of collection. To minimize enzymatic degradation and the loss of carotenoids and polyphenols due to oxidation, all handling steps were performed at a low temperature, and exposure to light and air was reduced. Following sample collection, the tomatoes were mechanically pressed to separate the juice from the solid fraction. The remaining by-product, peels, and seeds were collected, homogenized, and immediately stored in airtight polyethylene containers. Although the pomace was produced in laboratory conditions, the mechanical pressing and separation procedures replicate industrial-scale processing (pressing, separation, drying). Thus, compositional outcomes remain fully comparable to industrial by-products. To preserve their biochemical integrity, the samples were kept at −20 °C until further extraction and analysis. All handling steps were performed under cooled conditions to limit enzymatic degradation and oxidation of bioactive compounds.

### 3.2. Chemicals, Analytical Instrumentation, and Analytical Methods

#### 3.2.1. Reagent, Standard Solutions, and Certified Reference Materials (CRMs)

Chemicals and reagents of analytical grade or higher purity were used for analyses. Nitric acid ultrapure (60%), hydrogen peroxide for analysis (30%), boric acid for analysis, sulfuric acid for analysis (98%), chloroform for analysis, methanol for analysis ultrapure, potassium chloride (for analysis EMSURE^®^), sodium chloride (for analysis EMSURE^®^), sodium hydrogen sulfate monohydrate (extra pure), sodium sulfate (anhydrous for analysis EMSURE^®^), were obtained from Merck (Darmstadt, Germany). For metal determination, the calibration standard solutions were prepared from the 1000 mg L^−1^ multi-element IV ICP solution (Merck (Darmstadt, Germany) by dilutions in 2% HNO_3_. An ultrapure water (Elga Veolia, High Wycombe, United Kingdom) system was used for sample preparation. A certified reference material with a biological material matrix (cabbage powder), GBW10014, was purchased from the Institute of Geophysical and Geochemical Exploration, Langfang, China, and was analysed in parallel to check the trueness of the element’s determination. The obtained recoveries were in the range of 81 to 114%, indicating satisfactory performance for method trueness. Fatty acid methyl esters standard mixture (Supelco 37 components FAME mix, CRM47885) was purchased from Sigma-Aldrich (Merck KGaA, Darmstadt, Germany).

#### 3.2.2. Determination of Proximate Analysis of Tomato Pomace By-Products

The proximate analyses of tomato pomace were performed according to methodologies described in a previous work [53]. For moisture contents, samples were dried at 105 °C in an oven (UFE 400, Memmert, Schwabach, Germany) for 16 h, then measured by gravimetry. The protein content of the samples was calculated from the nitrogen content determined by the Kjeldhal method by measuring the nitrogen content released in 4% boric acid next to digestion with 98% H_2_SO_4_ and nitrogen conversion to protein with a factor of 4.38. The ash content was measured after sample decomposition at 600 °C in a Nebartherm Muffle Furnan (Nebartherm LT9/1300/C450, Lilienthal, Germany), by gravimetry. The fat content was obtained by gravimetry, after extraction of 3 g of dried samples with 50 mL chloroform/methanol (2:1, *v*:*v*) mixture for 60 min in an ultrasonic bath (ISOLAB, Schwabach, Germany), phase separation, drying, and weighing. Dietary fiber was analyzed by using Fourier Transform Near-Infrared Spectroscopy (FT-NIR) with a Tango instrument (Bruker Optics, Ettlingen, Germany) by scanning near-infrared spectra of the samples. Each sample was analyzed in triplicate, and results were expressed as grams per 100 g of dry matter (g 100 g^−1^, dry basis).

#### 3.2.3. Analysis of the Mineral Composition of Tomato Pomace

To determine mineral elements in tomato pomace, one gram of sample powder was mixed with 9 mL of HNO_3_ (60%) and 3 mL H_2_O_2_ (30%) in Berzelius beakers and kept at room temperature overnight (about 16 h). Subsequently the mixture was heated to reflux (about 120 ± 10 °C) for two hours, to obtain clear solutions. After cooling down at room temperature, the solutions were filtered through 0.45 μm filters (Whatman, Clifton, NJ, USA) and were diluted to 25 mL with ultrapure water. The concentrations of elements in digestion solutions were analyzed using an ICP-OES Optima 5300 DV spectrometer (Perkin Elmer, Ontario, Canada). Three replicates were analyzed for the determination of elements. Blanks and a solution with known concentrations of analytes (1 mg L^−1^) were also analyzed for quality control.

#### 3.2.4. Analysis of Fatty Acids Compositions

##### Extraction of Lipids from the Samples of Tomato Pomace and Fatty Methyl Esters (FAMEs)

Fatty acids were analyzed using gas chromatography with flame ionization detection (GC-FID) following the transesterification of lipids into fatty methyl esters after lipid extraction. The extraction method employed was the Bligh and Dyer method with adjustment, as previously described by Kalogeropoulos et al. [54].

##### Determination of Fatty Acid Methyl Esters (FAMEs) Content Using GC-FID

The content of fatty acid methyl esters (FAMEs) in tomato skin was determined using a gas chromatograph equipped with a flame ionization detector (GC-FID) (6890N, Agilent Technologies, Santa Clara, CA, USA) and a ZB-WAX capillary column (30 m × 0.25 mm × 0.25 µm) (Agilent Technologies, Santa Clara, CA, USA). Helium (6.0 purity, Linde Gaz, Cluj-Napoca, Romania) served as the carrier gas, maintained at a constant flow rate of 1 mL min^−1^. The split ratio was set at 1:20, and the injected volume was 1 µL. The temperature program for the GC oven comprised three stages: 60 °C for 1 min, a ramp from 60 to 200 °C at a rate of 10 °C min^−1^ for 2 min, and a subsequent ramp from 200 to 220 °C at 5 °C min^−1^ for 20 min. Both the injector and detector temperatures were maintained at 250 °C to ensure complete vaporization of the sample and optimal detection sensitivity. The retention times of the sample FAMEs were compared with those of a FAME standard mixture.

#### 3.2.5. Estimation of Volatile Compounds

Volatile compounds were analyzed using a gas chromatograph coupled with a mass spectrometer (GC-MS) (6890N, Agilent Technologies, Santa Clara, CA, USA) by using they HS-SPME-GC-MS technique. The system was equipped with an HP-5-MS capillary column (60 m length, 0.2 mm I.D., 0.25 µm film thickness) (Agilent Technologies, Santa Clara, CA, USA). A quantity of 3 g of tomato samples was introduced into a headspace vial, preceded by the addition of 3 g of NaCl to enhance volatility. The headspace vials were sealed with crimp-top caps that were fitted with TFE-silicone headspace septa. Compounds were identified based on a mass spectrum matching score of ≥80%. Results were expressed as the percentage of the relative peak area (% RPA) for each compound in the pepper samples, determined by dividing the individual peak area by the total peak area of all identified peaks in each chromatogram. For each sample, a total ion chromatogram (TIC) was used to perform peak area integration. Every measurement was performed three times, and the results were shown as the mean ± standard deviation.

#### 3.2.6. Determination of Total Polyphenolic Content (Folin–Ciocalteu Method)

A 0.5 g portion of the homogenized tomato by-product sample was extracted with 10 mL of methanol (MeOH) using a vortex mixer (Vortex 2, IKA, Staufen, Germany) at 2500 rpm for 2 min. The resulting mixture was centrifuged at 11,000 rpm for 2 min, and the supernatant was filtered through a 0.45 µm cellulose membrane filter. An adapted method based on Bobková et al. [55] was used for polyphenol quantification. In a 15 mL centrifuge tube, the reaction mixture consisted of 5 mL of distilled water, 1.5 mL of 10% sodium carbonate solution, 0.5 mL of filtered extract, and 0.5 mL of Folin–Ciocalteu reagent. The samples were incubated in the dark at room temperature for 45 min. Absorbance was measured at 765 nm using a spectrophotometer (Lambda 25, PerkinElmer, Waltham, MA, USA). Results were expressed as gallic acid equivalents (GAE). All analyses were performed in triplicate to ensure precision and reproducibility.

#### 3.2.7. Determination of Total Antioxidant Capacity

The antioxidant capacity of the samples was evaluated using a PHOTOCHEM chemiluminescence analyzer (Analytik Jena, Jena, Germany) in conjunction with the ACL assay kit. For extraction, 0.5 g of ground tomato by-product (pomace) was mixed with 1 mL of methanol and subjected to ultrasonic extraction for 20 min at room temperature. The extract was then centrifuged at 11,000 rpm for 2 min, and the supernatant was filtered through a 0.45 µm cellulose membrane filter. The resulting methanolic extract was directly injected into the analyzer. Antioxidant capacity was determined based on the extract’s ability to scavenge superoxide radicals and was expressed as milligrams of Trolox equivalents per gram of dry sample (mg TE g^−1^ DW). All measurements were performed in triplicate to ensure analytical reproducibility. All analyses were performed in triplicate to ensure precision and reproducibility.

#### 3.2.8. Determination of Total Carotenoids

The total carotenoid content was determined by spectrophotometric analysis following hexane extraction. A 20 g portion of homogenized tomato by-product (pomace) was transferred into a plastic container and extracted with 25 mL of hexane. The mixture was homogenized using a Stomacher 80 Biomaster (Seward, UK) for 120 s at room temperature to ensure efficient extraction of lipophilic carotenoids. After homogenization, the extract was filtered to remove solid residues, and the clear solution was transferred into quartz cuvettes for spectrophotometric analysis. Absorbance was measured at 450 nm using a UV-Vis spectrophotometer (Lambda 25, PerkinElmer, Waltham, MA, USA), and total carotenoid content was quantified based on a β-carotene calibration curve. Values were expressed as milligrams of β-carotene equivalents per gram of dry sample. All analyses were performed in triplicate to ensure precision and reproducibility.

#### 3.2.9. HPLC DAD Determination of Lycopene and β-Carotene

Lycopene and β-carotene were extracted from 0.5 g of homogenized dried tomato by-product (pomace) using 10 mL of a solvent mixture composed of hexane:acetone:ethanol (2:1:1, *v*/*v*/*v*). The mixture was vortexed thoroughly and allowed to stand at room temperature for 10 min to facilitate pigment solubilization. Following extraction, the samples were centrifuged at 11,000 rpm for 2 min, and the supernatant was filtered through a 0.45 µm cellulose membrane filter prior to HPLC analysis. All steps were conducted under reduced light conditions to minimize carotenoid degradation. Lycopene and β-carotene concentrations were determined using a UHPLC system (Vanquish, Thermo Fisher Scientific, Germering, Germany) equipped with a diode array detector (DAD). Chromatographic separation was performed on a Thermo Scientific™ Acclaim™ C30 column (3 µm, 3.0 × 150 mm) maintained at 40 °C. The mobile phase consisted of methanol containing 3.2 g L^−1^ ammonium acetate (70%) and acetonitrile (30%), delivered at a flow rate of 1.7 mL/min. An injection volume of 8 µL was used for all analyses. Detection was carried out at 460 nm, a wavelength suitable for both lycopene and β-carotene. Quantification was based on an external calibration curve prepared using certified HPLC standards of lycopene and β-carotene (Supelco, St. Louis, Sigma-Aldrich), with four concentration points: 1, 5, 10, and 20 ng mL^−1^.

#### 3.2.10. Nutritional Quality Indices and Calculation Formulas

Quality indices were calculated based on FAMES content according to Dongmo et al. [50]. The following indices were analyzed: atherogenic index (AI), thrombogenic Index (TI), hypocholesterolemic/hypercholesterolemic ratio (h/H), health-promoting index (HPI), nutritive value index (NVI), polyene index (PI), and desirable fatty acids (DFA). Equations (1)–(7) used for quality indices calculation are presented in Table 7.

#### 3.2.11. Statistical Analyses

The statistical differences in the studied parameters were evaluated by comparing the averages of the three replicates using Tukey’s test (*p* = 0.05) using Paired Comparison App (Two-Way Analysis of Variance (ANOVA)) by using Origin software (version 2020b, OriginLab, Northampton, MA, USA). The letters a, b, and c indicate statistically significant differences at *p* < 0.05. Pearson correlation and multivariate analysis were performed using Minitab for Windows, version 17.0 (Minitab, LLC, State College, PA, USA), to evaluate the relationships among the nutritional components of the tomato cultivars. Correlation coefficients were considered significant at *p* < 0.05. Multivariate analysis, principal component analysis (PCA), and hierarchical clustering, were applied to identify patterns of variation and groupings among the cultivars based on their chemical profiles.

## 4. Conclusions

The findings of this study demonstrate that tomato pomace is a valuable by-product with significant nutritional and functional potential for use as an ingredient in food and feed applications. A total of 57 volatile compounds were identified across the three cultivars, with their distribution varying by variety. These compounds included hydrocarbons, terpenoids, aldehydes, ketones, esters, alcohols, nitrogenous compounds, amines, amides, nitriles, isocyanates, pyridine, and pyrazine, all of which contribute to the aromatic profile of the samples. Mediterranean cultivars were characterized by higher levels of alcohols, hydrocarbons, and aldehydes, whereas Romanian cultivars exhibited greater amounts of terpenes, hydrocarbons, and aldehydes, enhancing their sensory properties. Mineral analysis revealed that all samples contained essential elements, including potassium, phosphorus, calcium, magnesium, sodium, iron, zinc, manganese, and copper. Magnesium was present in particularly high amounts across all cultivars and is known to play a critical role in numerous physiological functions. The samples also exhibited high levels of polyunsaturated fatty acids, especially linoleic acid, which are associated with cardiovascular health benefits. The manuscript integrates proximate composition, fatty acid profiles, volatile compounds, mineral content, and bioactive compounds. This comprehensive approach is rarely applied simultaneously to this agricultural by-product and offers novel insights into its valorization potential. The findings contribute to advancing sustainable practices and improving resource efficiency.

The present study utilized conventional solvent-based extraction methods, which are associated with environmental and safety constraints. Future research should therefore prioritize the development of green extraction strategies that reduce or eliminate chemical solvents, thereby improving both safety and environmental sustainability while enhancing the industrial applicability of tomato pomace in accordance with current sustainability objectives.

These compositional features are supported by favorable nutritional quality indices, including low atherogenic and thrombogenic values and high proportions of hypocholesterolemic fatty acids. Collectively, these results endorse the sustainable valorization of tomato processing residues as functional ingredients, thereby contributing to a circular bioeconomy and the development of health-oriented food products.

## Figures and Tables

**Table 1:** Proximate analysis (mean ± SD) of tomato pomace by-products from three tomato (*Solanum lycopersicum* L.) cultivars. Values are expressed as percentage (%) on fresh weight basis.

Code	Dry Matter	Fat	Protein	Ash	Dietary Fiber	Carbohydrates	Total Sugars
R1	33.31 ± 1.36 ^b^	2.4 ± 0.25 ^a^	6.66 ± 0.33 ^b^	0.95 ± 0.09 ^b^	7.97 ± 0.71 ^b^	32.7 ± 3.30 ^a^	5.1 ± 0.52 ^b^
R2	33.14 ± 1.44 ^ab^	1.9 ± 0.16 ^b^	8.43 ± 0.69 ^a^	0.88 ± 0.11 ^b^	7.27 ± 0.71 ^b^	26.0 ± 2.36 ^b^	6.7 ± 0.61 ^a^
R3	34.74 ± 1.90 ^a^	1.7 ± 0.17 ^c^	7.14 ± 0.36 ^b^	1.33 ± 0.14 ^a^	9.25 ± 0.85 ^a^	26.5 ± 2.74 ^b^	5.0 ± 0.55 ^b^

Note: Data are expressed as mean ± standard deviation (n = 9). The different letters indicate significant differences (*p* < 0.05) based on Tukey’s test.

**Table 2:** The average contents of mineral elements in tomato pomace (mg kg^−1^ dw) (average ± SD).

Element	R1	R2	R3
P	2980 ± 233 ^a^	2461 ± 185 ^a^	2990 ± 310 ^a^
K	11,940 ± 1020 ^a^	13,130 ± 1220 ^a^	14,200 ± 1230 ^a^
Ca	1210 ± 142 ^a^	1185 ± 173 ^a^	969 ± 145 ^a^
Mg	765 ± 110 ^a^	981 ± 137 ^a^	890 ± 110 ^a^
Na	166 ± 18 ^b^	385 ± 45 ^a^	224 ± 22 ^b^
Fe	19.2 ± 2.2 ^a^	10.1 ± 1.8 ^b^	7.8 ± 1.1 ^b^
Mn	6.30 ± 0.71 ^ab^	5.79 ± 0.66 ^b^	7.86 ± 0.79 ^a^
Cu	3.52 ± 0.40 ^a^	2.69 ± 0.36 ^b^	3.03 ± 0.45 ^ab^
Zn	10.3 ± 1.7 ^b^	10.5 ± 1.2 ^ab^	13.4 ± 2.0 ^a^

Note: Data are expressed as mean ± standard deviation (n = 9). The different letters indicate significant differences (*p* < 0.05) based on Tukey’s test.

**Table 3:** The ranges and concentrations of fatty acids (average ± SD) determined in tomato pomace varieties are presented as % of total acid content.

Type of Acids		R1	R2	R3
myristic acid	C14:0	0.30 ± 0.03 ^b^	0.21 ± 0.020 ^c^	0.38 ± 0.021 ^a^
palmitic acid	C16:0	14.89 ± 0.87 ^a^	10.19 ± 0.60 ^b^	13.72 ± 0.80 ^a^
palmitoleic acid	C16:1(n7)	0.28 ± 0.016 ^a^	0.24 ± 0.014 ^a^	0.31 ± 0.018 ^a^
heptadecanoic acid	C17:0	1.08 ± 0.063 ^a^	0.88 ± 0.084 ^ab^	0.83 ± 0.08 ^b^
stearic acid	C18:0	8.41 ± 0.85 ^a^	10.19 ± 1.03 ^a^	9.09 ± 0.92 ^a^
cis + trans-oleic acid	C18:1(c + t)(n9)	11.09 ± 1.13 ^b^	14.58 ± 1.48 ^ab^	17.05 ± 1.73 ^a^
cis + trans-linoleic acid	C18:2(c + t)(n6)	55.14 ± 5.62 ^a^	50.26 ± 5.12 ^a^	45.19 ± 4.60 ^b^
gama-linolenic acid	C18:3(n6)	3.05 ± 0.31 ^b^	4.15 ± 0.42 ^a^	4.75 ± 0.48 ^a^
α-linolenic acid	C18:3(n3)	2.09 ± 0.21 ^a^	2.48 ± 0.25 ^a^	2.66 ± 0.27 ^a^
arachidic acid	C20:0	0.23 ± 0.023 ^c^	0.32 ± 0.032 ^b^	0.41 ± 0.042 ^a^
eicosadienoic acid	C20:2(n6)	2.03 ± 0.20 ^c^	4.04 ± 0.41 ^a^	3.15 ± 0.32 ^b^
behenic acid	C22:0	1.00 ± 0.10 ^b^	2.25 ± 0.22 ^a^	2.12 ± 0.20 ^a^
lignoceric acid	C24:0	0.42 ± 0.040 ^a^	0.22 ± 0.021 ^b^	0.35 ± 0.035 ^a^
Saturated fatty acids	SFA	11.44 ± 1.16 ^a^	14.07 ± 1.43 ^a^	13.17 ± 1.34 ^a^
Monounsaturated fatty acids	MUFA	25.98 ± 2.64 ^a^	24.77 ± 2.52 ^a^	30.77 ± 3.13 ^a^
Polyunsaturated fatty acids	PUFA	62.30 ± 6.35 ^a^	60.92 ± 6.21 ^a^	55.75 ± 5.68 ^a^
	omega 6	57.17 ± 6.5 ^a^	54.29 ± 5.53 ^a^	48.34 ± 4.93 ^a^
	omega 3	2.09 ± 0.21 ^a^	2.48 ± 0.25 ^a^	2.66 ± 0.27 ^a^
	UFA	88.28 ± 9.0 ^a^	85.69 ± 8.74 ^a^	86.52 ± 8.82 ^a^

Note: The different letters indicate significant differences (*p* < 0.05) between the average results for each component.

**Table 4:** The volatile compounds found in tomato pomace waste. The values presented indicate the peak area of each compound (%) (average ± standard deviation (SD).

Nr. Crt.	Retention Time (min)	Compound Name	R1	R2	R3	Sensory Descriptors
Hydrocarbon						
1.	2.793	1,5-Hexadiyne	1.00 ± 0.12 ^a^	ND	ND	Slightly sweet and mildly pungent
2.	3.006	3-Methyl-2,4-hexadiene	0.90 ± 0.091 ^b^	ND	7.39 ± 0.75 ^a^	Green, resinous, and slightly sweet
3.	3.019	4-Methylcyclohexene	ND	3.43 ± 0.28 ^a^	ND	Mildly sweet, and less fragrant
4.	8.717	5-chloro -methylpenta-1,3-diene	ND	5.40 ± 0.34 ^a^	ND	Green, herbaceous, and slight sweet
5.	8.736	3-azidoprop-1-ene	ND	ND	1.76 ± 0.12 ^a^	Pungent, acrid, and irritant
6.	8.930	trans-1,4-Hexadiene	ND	ND	19.78 ± 1.24 ^a^	Light green, slight sweet
7.	9.374	1,3-Butadiene	ND	0.12 ± 0.02 ^a^	ND	Mildly sweet and minor green
8.	9.718	2-Vinylbicyclo [2.1.1]hex-2-ene	ND	0.38 ± 0.028 ^a^	ND	Slight sweet, light, and resinous notes
9.	9.781	2,4-Octadiyne	ND	0.25 ± 0.021 ^a^	ND	Slightly metallic, pungent
10.	10.187	Propylcyclopropane	6.70 ± 0.58 ^a^	7.62 ± 0.71 ^a^	ND	Hydrocarbon odor, mild
11.	10.737	1-Butene	0.02 ± 0.01 ^b^	0.06 ± 0.005 ^b^	0.78 ± 0.08 ^a^	Light hydrocarbon odor, slight sweet
12.	12.383	(E)-1,2-Dimethylcyclopropane	0.13 ± 0.01 ^a^	ND	0.03 ± 0.0003 ^a^	Light hydrocarbon odor and slight sweet
13.	13.934	1,5-Heptadien-3-yne	ND	1.29 ± 0.14 ^a^	ND	Sharp, pungent, and green
14.	15.936	3,7,7-Trimethylcyclohepta-1,3,5-triene	ND	1.72 ± 0.15 ^a^	ND	Pine, fresh, and woody
15.	16.486	1-Pentene	0.05 ± 0.004 ^a^	ND	ND	Light hydrocarbon odor, slight sweet
16.	17.844	2-methyl-1,3-pentadiene	ND	0.07 ± 0.007 ^a^	ND	Greem, herbaceous, and middle fruity
17.	18.438	3-Methyl-4-methylenebicyclo(3.2.1)oct-2-ene	ND	0.11 ± 0.012 ^a^	ND	Woody, resinous, and herbal
18.	19.895	N-Methylene-ethenamine	0.05 ± 0.004 ^a^	ND	ND	Sharp, pungent amine odor
19.	21.640	1-Fluoroheptane	0.42 ± 0.04 ^a^	ND	ND	Low odor
Nitrogen Compounds						
20.	2.699	2-Nitrophenyl azide	0.24 ± 0.02 ^a^	ND	ND	Pungent and slightly sweet
21.	15.473	3-Azido-1-propene	0.03 ± 0.002 ^a^	ND	ND	Sharp, pungent
Terpenes						
22.	5.777	Prenol	ND	4.74 ± 0.32 ^a^	ND	Green, fruity, floral, and fresh
23.	17.299	Terpinolene	ND	20.05 ± 1.98 ^a^	ND	Fresh, herbal, and green
24.	17.625	Limonene	ND	1.57 ± 0.14 ^a^	ND	Citrus, sweet, fresh, and fruity
25.	18.106	Ocimene	ND	0.52 ± 0.03 ^a^	ND	Sweet, fresh, green, and slight citrus
26.	18.638	Beta-phellandrene	ND	47.23 ± 4.1 ^a^	ND	Minty, light citrus, green and herbal
27.	29.272	Cholestanone	ND	ND	0.47 ± 0.05 ^a^	Slight waxy
Alcohols						
28.	3.875	1-Octen-3-ol	1.48 ± 0.15 ^b^	1.93 ± 0.087 ^a^	ND	Waxy, fatty, green, and light citrus
29.	8.917	2-methyl-1-propanol	4.24 ± 0.32 ^a^	0.70 ± 0.06 ^c^	1.76 ± 0.13 ^b^	Green, herbaceous, fruity, and fresh
30.	10.212	1-Hexanol	ND	ND	30.91 ± 2.87 ^a^	Fatty, waxy, green, herbaceous
Aldehydes						
31.	5.783	Hexanal	6.96 ± 0.71 ^a^	ND	1.76 ± 0.01 ^b^	Green, herbal, and fresh notes
32.	8.711	2-Hexenal	9.98 ± 0.87 ^b^	ND	19.63 ± 1.2 ^a^	Green, fruity, herbal, and sharp
33.	10.512	Acrolein	0.16 ± 0.01 ^b^	0.03 ± 0.002 ^c^	0.66 ± 0.05 ^a^	Pungent, acrid
34.	15.648	2-Ethylhexenal	0.09 ± 0.008 ^a^	ND	ND	Green, fruity, citrusy, and slight sweet
35.	17.056	Pent-2-ynal	ND	0.30 ± 0.02 ^a^	ND	Sharp, green, fresh, and metallic
Ketones						
36.	4.682	Methylvinylketone	0.10 ± 0.01 ^b^	ND	5.98 ± 0.42 ^a^	Pungent, acrid, unpleasant
37.	16.861	6-Methyl-5-heptene-2-one	2.20 ± 0.18 ^a^	ND	ND	Fruity, green, and fresh
38.	39.118	Cyclohexane-1,3-dione, 2-allylaminomethylene-5,5-dimethyl	0.03 ± 0.002 ^c^	0.15 ± 0.01 ^b^	0.89 ± 0.07 ^a^	Fresh, green, and herbal nuances
Amines						
39.	4.507	Aminooxyacetic Acid	0.16 ± 0.01	0.21 ± 0.01	0.04 ± 0.002	Pungent, acidic, and amine-like odors
40.	6.065	Allylamine	0.07 ± 0.006 ^a^	ND	0.005 ± 0.0004 ^b^	Ammoniacal, fishy, sharp, pungent
41.	9.649	2-Amino-1,3-propanediol	0.08 ± 0.007 ^a^	ND	0.08 ± 0.006 ^a^	Slight sweet
42.	10.888	2-Propyn-1-amine	0.01 ± 0.001 ^a^	ND	ND	Sharp, pungent, and irritant
43.	19.220	2,5-Norbornadiene	0.12 ± 0.01 ^a^	ND	ND	Light sweet, fresh, and herbal nuances
44.	40.163	Methallylamine	ND	ND	0.62 ± 0.52 ^a^	Pungent, acrid, and ammoniacal notes
Nitriles						
45.	9.305	2-Propenenitrile	0.06 ± 0.008 ^b^	ND	0.15 ± 0.02 ^a^	Acrid, sharp, pungent, and irritating
46.	9.874	Propiolonitrile	ND	0.14 ± 0.01 ^a^	ND	Acrid, almond like
Amides						
47.	18.788	N-Ethyl-N-methylacetamide	0.80 ± 0.07 ^b^	ND	4.28 ± 0.24 ^a^	Slight sweet
48.	18.801	Acrylamide	ND	0.32 ± 0.02 ^a^	ND	Slight acrid, plastic like
Pyrazines						
49.	16.887	2,3-Dimethylpyrazine	ND	0.59 ± 0.06 ^b^	0.63 ± 0.04 ^a^	Fruity, sweet, and green
50.	38.574	2-Methyl-3-(2-propenyl)pyrazine	0.18 ± 0.01 ^a^	ND	ND	Nutty and roasted
Pyridines						
51.	35.752	2-Methyl-3-(prop-2-ynyloxy)pyridine	0.10 ± 0.01 ^a^	ND	ND	Mildly acrid and slight bitter
52.	36.278	1H-Dipyrido [2,3-b:3′,2′-d]pyrrole	0.01 ± 0.001 ^a^	ND	ND	Sharp, pungent nitrogenous odor
53.	39.574	2-Methyl-3-[(prop-2-yn-1-yl)oxy]pyridine	0.14 ± 0.01 ^a^	ND	ND	Pungent, herbal
Esters						
54.	15.636	Methylthiophosphonamidic acid, S-methyl ester	ND	ND	0.04 ± 0.005 ^a^	Pungent, garlic-like, and acrid
Heterocyclic						
55.	2.825	Azetidine	ND	1.26 ± 0.12 ^a^	1.09 ± 0.11 ^a^	Ammoniacal, fishy, pungent
Furans						
56.	17.049	2-Pentylfuran	0.72 ± 0.05 ^b^	ND	1.27 ± 0.15 ^a^	Green, grassy, nutty, and woody
Isocyanates						
57.	17.693	Methyl isocyanate	0.06 ± 0.004 ^a^	ND	ND	Sharp, pungent, acid

Note: The different letters indicate significant differences (*p* < 0.05) between the average results for each component. ND-not determined.

**Table 5:** Bioactive compound content and antioxidant capacity of tomato pomace by-products from three tomato (*Solanum lycopersicum* L.) cultivars. Values are expressed as mean ± standard deviation.

Sample Name	Total Polyphenols (mg GAE.kg^−1^)	Antioxidant Capacity (µg eq Trolox. g^−1^ )	Total Carotenoids (mg eq. β-carotene. g^−1^)	β-Carotene (µg g^−1^)	Lycopene (µg g^−1^)
R1	134.71 ± 12.3 ^ab^	0.34 ± 0.03 ^a^	4.24 ± 0.40 ^a^	2.95 ± 0.27 ^b^	6.22 ± 0.63 ^b^
R2	122.03 ± 10.0 ^b^	0.31 ± 0.03 ^b^	2.24 ± 0.22 ^c^	3.10 ± 0.29 ^a^	6.10 ± 0.56 ^b^
R3	133.85 ± 11.5 ^a^	0.34 ± 0.03 ^a^	2.97 ± 0.27 ^b^	3.14 ± 0.30 ^a^	7.41 ± 0.68 ^a^

Note: Data are expressed as mean ± standard deviation (n = 3). Values indicated with different letters were significantly different from each other at *p* ≤ 0.05 levels, whereas the same letters showed no significant differences (*p* > 0.05). Different letters in each column showed a significant difference at the level of *p* ≤ 0.05.

**Table 6:** Nutritional quality indices.

Index	R1	R2	R3
MUFA/SFA	2.27	1.76	2.34
PUFA/SFA	5.45	4.33	4.23
$\frac{\sum PUFA(n-3)}{\sum PUFA(n-6)}$	0.037	0.046	0.055
$\frac{omega\text{-}6}{omega\text{-}3}$	27.35	21.89	18.17
TI	0.10	0.09	0.11
AI	0.18	0.13	0.18
h/H	6.54	9.64	7.34
HPI	5.81	8.24	6.14
NVI	69.62	103.19	84.30
PI	5.45	4.33	4.23
DFA	96.7	95.9	95.6

Note: MUFA—monounsaturated fatty acids; PUFA—polyunsaturated fatty acids; SFA—saturated fatty acids; AI—Atherogenic index; TI—Thrombogenic Index; h/H—Hypocholesterolemic/hypercholesterolemic index; HPI—Health-promoting index; NVI—nutritive value index; PI—peroxide index; and DFA—desired fatty acids.

**Table 7:** Nutritional quality indices and calculation formulas.

Parameter	Calculation Formulas	
TI ^a^	$\frac{\left(C14\text{:}0+C16\text{:}0+C18\text{:}0\right)}{[(0.5\times \sum UFA)\;+\;(0.5\times \sum PUFA\;n-6)+(3\times \sum PUFA\;n-6)+(\sum PUFA\;n-3/\sum PUFA\;n-6)}$	(1)
AI ^b^	$\frac{\left[C12\text{:}0+\left(4\times C14\text{:}0\right)+C16\text{:}0\right]}{\sum UFA}$	(2)
h/H ^c^	$\frac{(cis-C18\text{:}1+\sum MUFA+\sum PUFA}{\left(C12\text{:}0+C14\text{:}0+C16\text{:}0\right)}$	(3)
HPI ^d^	$\frac{\sum UFA}{\left[C12\text{:}0+(4\times C14\text{:}0)+C16\text{:}0\right]}$	(4)
NVI ^e^	$\frac{\left(C18\text{:}0+C18\text{:}1\right)}{C16\text{:}1}$	(5)
PI ^f^	$\frac{\sum PUFA}{\sum SFA}$	(6)
DFA ^g^	C18:0 + ∑UFA	(7)

^a^ Thrombogenic index; ^b^ atherogenic index; ^c^ hypocholesterolemic/hypercholesterolemic ratio; ^d^ health-promoting index; ^e^ nutritive value index; ^f^ polyene index; ^g^ desirable fatty acids.

## Data Availability

The original contributions presented in the study are included in the article.

## Abbreviations

The following abbreviations are used in this manuscript:

PUFA	Polyunsaturated fatty acid
MUFA	Monounsaturated fatty acids
SFA	Saturated fatty acids
K	Potassium
Ca	Calcium
P	Phosphorus
Mg	Magnesium
Fe	Iron
Cu	Cooper
Zn	Zinc
Na	Sodium
Mn	Manganese
B	Boron
Cd	Cadmium
Pb	Lead
Hg	Mercury
As	Arsenic
Al	Aluminum
GC-FID	Gas chromatograph equipped with a flame ionization detector
FAMEs	Fatty acid methyl esters
GC-MS	Gas chromatograph coupled with a mass spectrometer
RPA	Relative peak area
TIC	Total ion chromatogram
GAE	Gallic acid equivalents
TE	Trolox equivalents
DAD	Diode array detector
AI	Atherogenic index
TI	Thrombogenic Index
h/H	Hypocholesterolemic/hypercholesterolemic ratio
HPI	Health-promoting index
NVI	Nutritive value index
PI	Polyene index
DFA	Desirable fatty acids
ATP	Adenosine triphosphate
LDL	Low-density lipoproteins
UFA	Unsaturated fatty acids
LA	Linoleic acid
GLA	Gama-linolenic acid
